# Data for photoluminescence spectra of natural Cr^3+^-doped MgAl_2_O_4_ spinel during order-disorder transition

**DOI:** 10.1016/j.dib.2020.106310

**Published:** 2020-09-15

**Authors:** Chengsi Wang, Andy H. Shen, Yungui Liu

**Affiliations:** a Gemmological Institute, China University of Geosciences (Wuhan), Hubei Province 430074, China; b College of Gems and Materials Technology, Hebei GEO University, Hebei Province 050031, China

**Keywords:** Photoluminescence spectroscopy, Spinel, Order-disorder transition, Gemology, Thermal history

## Abstract

Photoluminescence (PL) spectra of natural Cr^3+^ doped MgAl_2_O_4_ spinel from Tanzania were taken during its order-disorder transition (ODT) process. Samples were changed their disordered degree by quenching treatment. PL spectra were taken at the liquid nitrogen (LN) temperature (∼77 K) using a 532 nm laser excitation. Spectra from different states were compared with each other and R-line and N-line ratio was used to illustrate the disordered degree of spinel during ODT process. It can be used as a reference for other similar researches, such as spinel PL characterization of the other members of this group, ordered degree of synthetic spinel single crystal or ceramics materials, thermal history of spinel, and non-destructive identification of natural and heated spinel gemstones, spinel original distinguishing, further PL spectra analysis and XRD relationship research.

## Specifications Table

**Table:** 

Subject	Geology
Specific subject area	Gemological material science
Type of data	Table Figure Txt
How data were acquired	Raman Spectrometer
Data format	Raw
Parameters for data collection	Controlled heating experiments were performed in an alumina tube furnace (HF-Kejing GSL-1700X), and placing the samples in an alumina crucible. All experiments were completed in the air and at atmospheric pressure. The sample was heated up to the target temperatures using a ramp rate of 2 °C /min. Recorded temperature of the tube furnaces was calibrated and corrected to an accuracy of +/- 1.5 °C.
Description of data collection	All the Photoluminescence (PL) spectra were taken at the liquid nitrogen (LN) temperature (∼77 K). The PL spectra were taken by a JY HORIBA LabRAM HR Raman spectrometer at the Gas Hydrate Evaluation Laboratory of Faculty of Earth Resource of China University of Geosciences using a 532.06 nm (Frequency doubled ND: YAG) laser excitation with a 10 × objective and an 1800-groove/mm grating. The output laser power is 45 mW with a 4D filter which made the actual laser power 0.0045 mW. The aperture of the confocal pinhole is 100 μm. Raman peak shift was calibrated regularly with the 520.7 cm^−1^ band of a polished silicon wafer. PL spectra were collected with exposure time, 2 s; accumulated 15 times; and resolution, 0.35–0.63 cm^−1^.
Data source location	Institution: China University of Geoscience City/Town/Region: Wuhan Country: China
Data accessibility	With the article

## Value of the Data

• The data report the PL spectra variation of Cr-doped MgAl_2_O_4_ spinel in order-disorder transition. Those who are interested the usage of PL spectra as a local probe for order-disorder transition could see the fine variations from this data. These data are also good references for studies of spinel PL characterization of the other members of this group, ordered degree of synthetic spinel single crystal or ceramics materials, and thermal history of spinel.
• Researchers who are interested in gemstone original distinguishing may want to see these data in details. These data were collected from spinel originating from Morogoro, Tanzania. Different with spinels from Burma, Vietnam, and Sir Lanka, the samples emitted very rare four R peaks (which usually only be two), and very sharp N peaks which is a good reference for comparison.
• The sharpness of the spectra makes the peaks are easily separated by peak-fitting software. For the future researchers who discover the structure-defect source of one specific PL peak, these data are good material for data analysis.
• The samples can also be compared with the XRD data trying to reveal the relationship between the local order-disorder transition and the long-range one. With the development of PL spectroscopy, once the N peaks assignments were explored, the data can be used as a good resource to reveal the atoms transition around the Cr ions.

## 1 Data Description

**Fig. 1:**
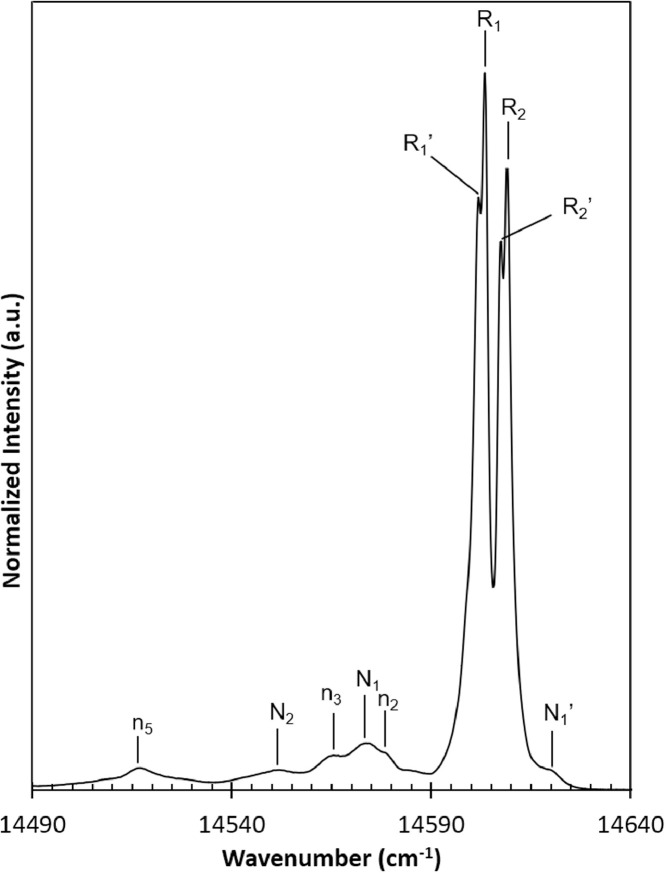
PL spectra of unheated natural MgAl_2_O_4_ spinel.

The PL spectrum of gem-quality MgAl_2_O_4_:Cr^3+^ spinel, originning from Tanzania, was collected and reported in details, presenting in raw data T1-unheated (Fig. 1). The PL spectrum of natural MgAl_2_O_4_ spinel consisted of two main parts: zero-phonon lines (ZPL) and phonon-sidebands (PSB), located in the range of 14640 cm^−1^ to 14490 cm^−1^ and 14490 cm^−1^ to 13700 cm^−1^ respectively. In ZPL range, four R lines and five N lines were obtained, their position were listed in the Table 1.

**Table 1:** Position of Zero-phonon peaks

	Peak Position (cm^−1^)
R_2_	14609.2
R_2_’	14607.6
R_1_	14603.4
R_1_’	14602.0
N_1_	14574.1
n_3_	14565.5
N_2_	14551.9
n_5_	14519.0

**Fig. 2:**
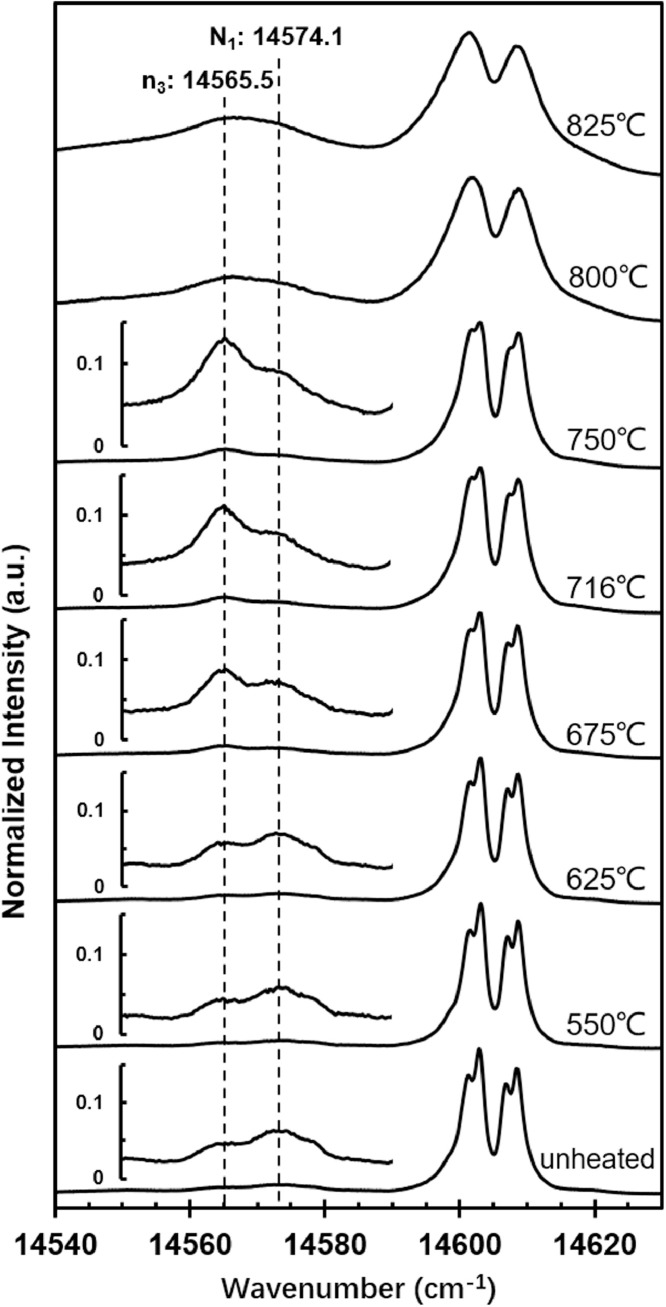
PL spectra of samples quenched from low temperatures.

In the temperature range 0–825 °C, the R peaks were the strongest peak of PL spectra. With the temperature increasing, R_1_, R_1_’, R_2_, and R_2_’ peaks became broad and finally merged into two peaks. In this process, the n_3_ peak gradually increased, but the N_1_ almost remained unchanged, presenting in raw data T1-550 ∼ 825 (Fig. 2).

**Fig. 3:**
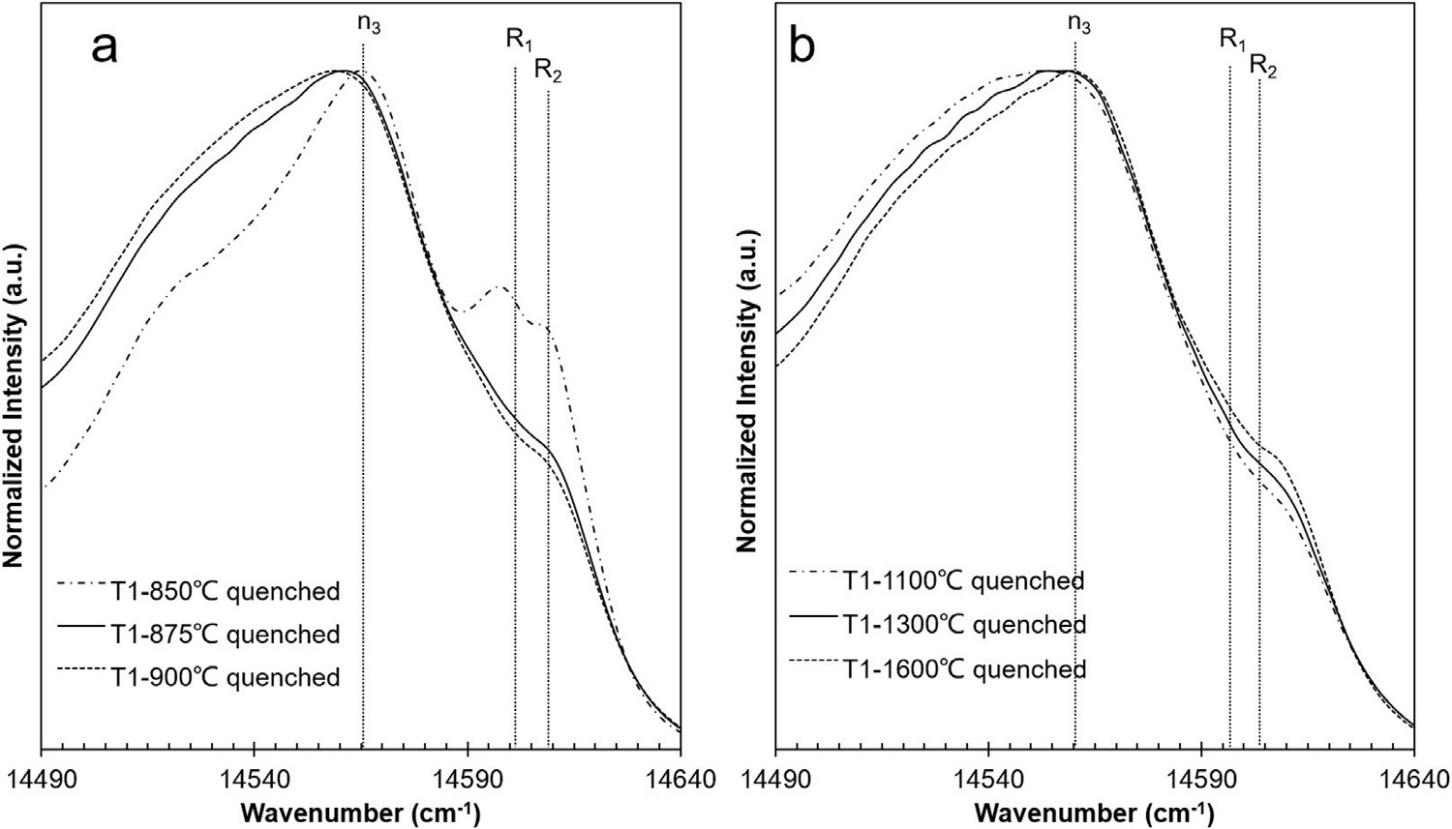
PL spectra of samples quenched from high temperatures.

At high temperature (850–1600 °C), all the PL peaks broaden and merged into a band, and variations were hardly detectable. The maximum value of this band located in the N peak range, and the R peaks intensity decreased significantly showing as shoulders, presenting in raw data T1-850 ∼ 1600 (Fig. 3).

## 2 Experimental Design, Materials, and Methods

An octahedron-shaped natural spinel crystal (T1), from Morogoro, Tanzanian was used in this study. The sample is gem-quality, transparent and red color. The sample was cut into several doubly polished parallel plates perpendicular to the crystallographic direction of [111] with thickness approximately 1.6–1.9 mm.

All the Photoluminescence (PL) spectra were taken at the liquid nitrogen (LN) temperature (∼77 K). The PL spectra were taken by a JY HORIBA LabRAM HR Raman spectrometer at the Gas Hydrate Evaluation Laboratory of Faculty of Earth Resource of China University of Geosciences using a 532.06 nm (Frequency doubled ND: YAG) laser excitation with a 10 × objective and an 1800-groove/mm grating. The output laser power is 45 mW with a 4D filter which made the actual laser power 0.0045 mW. The aperture of the confocal pinhole is 100 μm. Raman peak shift was calibrated regularly with the 520.7 cm^−1^ band of a polished silicon wafer. PL spectra were collected with exposure time, 2 s; accumulated 15 times; and resolution, 0.35–0.63 cm^−1^.

Controlled heating experiments were performed in an alumina tube furnace (HF-Kejing GSL-1700X), and placing the samples in an alumina crucible. All experiments were completed in the air and at atmospheric pressure. The sample was heated up to the target temperatures using a ramp rate of 2 °C/min. Recorded temperature of the tube furnaces was calibrated and corrected to an accuracy of +/- 1.5 °C.

Samples were heated directly to their target temperatures with no intermediate steps, held for 1 h, quickly pulled out from the furnace and quenched in the air.

## Related Research Article

Chengsi Wang, Andy H. Shen, Yungui Liu, Characterization of order-disorder transition in MgAl_2_O_4_:Cr^3+^ spinel using photoluminescence, Journal of Luminescence https://doi.org/10.1016/j.jlumin.2020.117552.

## Declaration of Competing Interest

The authors declare that they have no known competing financial interests or personal relationships which have, or could be perceived to have, influenced the work reported in this article.

## Appendix Supplementary Materials

Image, application 1

Image, application 2

