# Supplementary material for: Data for photoluminescence spectra of natural Cr3+-doped MgAl2O4 spinel during order-disorder transition
Source: Data Brief. 2020 Sep 15;32:106310. doi: 10.1016/j.dib.2020.106310 (PMC7511808; doi:10.1016/j.dib.2020.106310)
Supplement: Supplementary file 1 [file mmc1.docx]

**Credit Author statement**

**Chengsi Wang:** Investigation; Data Curation; Writing-Original Draft; Validation; Conceptualization; Funding acquisition

**Andy H Shen:** Conceptualization; Supervision; Project administration; Writing - Review & Editing; Funding acquisition

**Yungui Liu:** Resources; Investigation;
